# Supplementary material for: Strong coupling and high-contrast all-optical modulation in atomic cladding waveguides
Source: Nat Commun. 2017 Feb 9;8:14461. doi: 10.1038/ncomms14461 (PMC5309768; doi:10.1038/ncomms14461)
Supplement: Supplementary Information — Supplementary Figures, Supplementary Notes and Supplementary References. [file ncomms14461-s1.pdf]

Supplementary note 1

Transit time broadening contribution to the linear evanescent susceptibility

In this section we analyze numerically the susceptibility of atoms subjected to an evanescent field for the purpose of predicting the magnitude of transit time broadening and its dependence on the evanescent length. To do so we adapt the formalism of attenuated total internal reflection[1–4], which we have used in the main text for as a fitting model and has been found to yield good results in a variety of guided wave configurations[5–7]. For a single atomic transition the evanescent susceptibility is proportional to the following expression:

$$\chi(\nu) \sim \int_{-\infty}^{\infty} \int_{0}^{\infty} \frac{W(v_z, v_x)}{-i(\gamma - ik_z v_z) - (2\pi\Delta\nu - k_x v_x)} dv_x dv_z \qquad (1)$$

Where v_x and v_z are the atomic velocities in the propagation direction *x,* and the transverse direction (z) respectively, *W* is the two dimensional normalized velocity distribution, k_x and k_z represent the momentum of the evanescent field along the propagation direction and the transverse direction respectively. 2γ is the natural full-width half maximum line-width and Δv the frequency detuning from the transition frequency. The evanescent susceptibility includes two primary broadening mechanisms: Enhanced (relative to vacuum) Doppler broadening, and transit time broadening. For analyzing the transit time broadened line, one can integrate on the zero-velocity group in x direction, and obtain the following formula:

$$\chi(\nu) \sim \int_{0}^{\infty} \frac{W(v_z)}{-2\pi\Delta\nu - i(\gamma - ik_z v_z)} dv_z \qquad (2)$$

In figure supplementary 1 panels a-c we plot the normalized line shape (by integrating the above formula numerically) for three different evanescent lengths. One can clearly observe the cusp function character of the line-shape, which evolves with increasing the evanescent length into a Lorentzian. In figure supplementary 1d we plot the spectral width derived from Eq. 2 by applying a full width half maximum criteria and a full width at e^{-1} (we do so to better estimate the cusp-like behavior at small evanescent lengths). We compare this to the well established model[8], which relates the average velocity and the spot size to the spectral width by $\Delta\nu = \ln(2)\, v_{avg}/w$ where v_{avg} is the atomic one dimensional average velocity, and w is the spot diameter (in our case - the evanescent length). Clearly, all models agree for longer evanescent lengths, whereas for smaller (< 500nm) the ~1/w model over estimates the TTB, consistent with our observation in the main text.

We note, that as we are comparing one-dimensional models, we take the one dimensional average of the one sided Boltzmann distribution, namely $v_{avg} = \int_0^\infty v_z W(v_z) dv_z$ yielding an average of ~70m/s for the temperature of 65°C. Clearly, one can observe that Eq. 2 predicts a significantly smaller transit time broadened spectral width compared to inverse evanescent length model. For longer evanescent lengths the models agree well. Specifically, for the case depicted in the main text, where we have an evanescent length of ~100nm, we have a TTB FWHM of ~90MHz, and e^{-1} of 160 MHz.

Supplementary Figure 1:

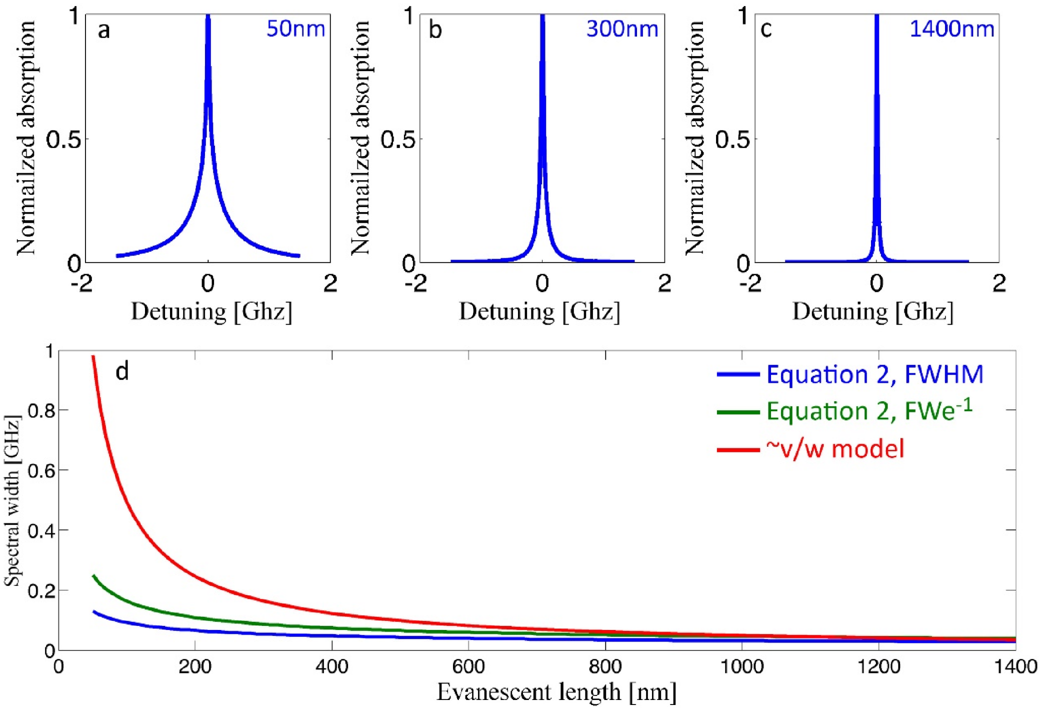

Supplementary Figure 1 | Transit time broadened theoretical line widths (a-c) Normalized absorption of v_z=0 group of atoms, in the case of attenuated total internal reflection, for the three different evanescent lengths a) 50nm b) 300 nm c) 1400nm. (d) Spectral width as function of evanescent length derived using Eq. 2 (blue and green) and the v/w model (red).

Supplementary note 2.

Van der Waals frequency shift calculation

In order to have a better theoretical estimation of the frequency shift associated with the long range van der Waals (VDW) shift induced by the waveguide surface, we incorporate the VDW shift within the framework of the non-local atomic response developed by Nienhuis et al. in ref. 1. Following this derivation within a susceptibility framework, it can be shown, that by separating the atoms into these two different groups one can write the following relation for the effective susceptibility of the atomic cladding:

$$\chi \sim \int_{0}^{\infty} dz \int dv W(v) e^{ik_z z} [\Theta(v_z)\sigma(z,v) + \Theta(-v_z)\bar{\sigma}(z,v)] \quad (3)$$

Where k_z is the imaginary z component wave number, W is the Maxwell-Boltzmann velocity distribution, $\sigma(z,v)$ and $\bar{\sigma}\,(z,v)$ are the coherences for out coming and incoming atoms, respectively, $\Theta(\mathrm{v})$ is the Heaviside function and **v** is the atomic velocity.

When solving the two level system, subject to an evanescent Rabi frequency one gets the following solutions for the incoming and out coming the off diagonal elements σ:

$$\sigma(z,v) \approx \frac{i\Omega(0)\cdot\left(e^{ik_z z} - e^{-\frac{z[\gamma - i(\Delta - k_x v_x)]}{v_z}}\right)}{2[\gamma - i(\Delta - k\cdot v)]} \qquad (4)$$

$$\bar{\sigma}(z,v) \approx \frac{i\Omega(0)\cdot e^{ik_z z}}{2[\gamma - i(\Delta - k\cdot v)]} \qquad (5)$$

Where 2γ is the natural full-width half maximum line-width, k is the guided wave number, Now, we can incorporate the VDW shift by replacing Δ with $\Delta + C/z^3$. The VDW coefficient C is assigned as $1.2KHz \cdot \mu m^3$ as reported for the $5S_{1/2}$ to $5P_{3/2}$ Rb transition with a sapphire window[9]. In figure supplementary 2 we plot the integrated steady state solution (i.e, the contribution of the incoming atoms to the susceptibility) with (blue line) and without (green line) the contribution of the VDW shift. Here we assume a 100MHz transit time broadened line (rather than integrating the v_z velocity group), an effective index of *1.66*. From this calculation, we find that the center of line shifts ~65MHz, which agrees well with the measured shift.

Supplementary Figure 2:

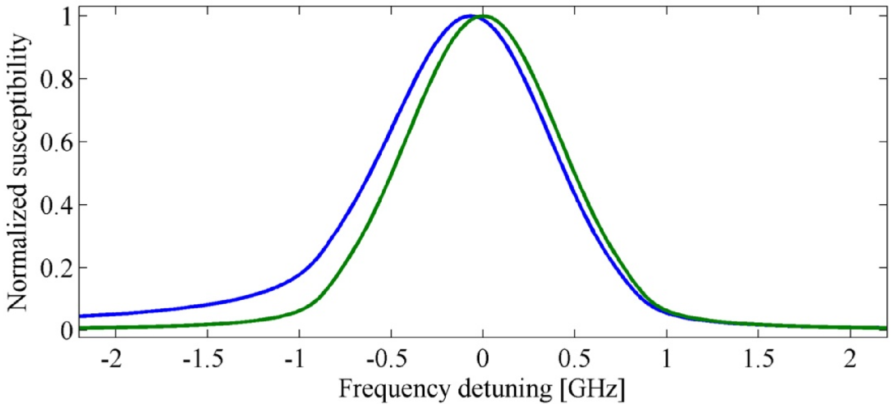

Supplementary Figure 2 | Van der Waals frequency shift in evanescent field: Calculation of the evanescent atomic susceptibility for incoming atoms, with (blue line) and without (green line) the of VDW shift.

Supplementary note 3.

V-Type Pump-probe evanescent susceptibility

Here, we calculate the effective susceptibility of atomic vapor subject to a one dimensional evanescent pump and probe electromagnetic distribution. To do so, we follow the general derivation given in references 1 and 10, and modify it to account for an evanescent pump-probe configuration in the V-type configuration. Herby, we shall briefly describe the model, and its results in context of the main manuscript.

In figure supplementary 3a we describe the V-type level scheme. In the case presented in the accompanied manuscript, the |1> to |2> transition and |1> to |3> transition correspond to the 780nm D2 and 795nm D1 lines respectively. In figure supplementary 3b we describe schematically the scenario we are modeling. A one dimensional slab wave guide is illustrated

as having a dielectric core, and an atomic vapor cladding. The electromagnetic mode that is supported by this wave guide, has a real wave number in direction x, and an imaginary wave number in the direction z. The later represents the evanescent wave. An atom is illustrated to have two distinct spatial-dynamic states: a) an atom with a *negative* velocity Vz (Vz is defined as positive in the direction of the z axis) entering the evanescent region. Such an atoms state will subsequently be described to be in steady state with a pump and probe that have an exponentially increasing electric field. Such a state is assumed to follow the spatial variation adiabatically, starting at the ground state far away from the surface. b) an atom with a *positive* Vz, which has left the surface at a quenched state, i.e, in it's ground state. Such an atom is subject to an abrupt change in its atomic state due to its collision with the surface (and consequently loss of coherence) in the presence of electromagnetic field, and thus the atom cannot be assumed to be in steady state.

It can be shown, that by separating the atoms into these two different groups one can write the following relation[1,4,10] for the effective susceptibility of the atomic cladding:

$$\chi = \frac{-2ik_z\hbar\Omega_s N}{I_s}\int_{\mathbf{0}}^{\infty} dz \int dv W(v) e^{ik_z z}[\Theta(v_z)\sigma_{13}(z,v) + \Theta(-v_z)\overline{\sigma_{13}}(z,v)] \quad (6)$$

Where k_z is the imaginary z component wave number (k is the wave number in free space), Ω_s, I_s are the Rabi frequency and intensity of the probe, respectively, N is the atomic density, W is the Maxwell-Boltzmann velocity distribution, $\sigma_{13}(z,v)$ and $\overline{\sigma_{13}}\,(z,v)$ are the probes coherences for out coming and incoming atoms, respectively and $\Theta(\mathrm{v})$ is the Heaviside function. Thus, in order to calculate the susceptibility we are in need to find both $\sigma_{13}(z,v)$ and $\overline{\sigma_{13}}(z,v)$. We start by writing the general Bloch equations, in the spatial domain[10–12]:

$$\begin{cases} v_z \frac{d}{dz}\sigma_{11} = \frac{i\Omega_p(z)}{2}(\sigma_{21}-\sigma_{12}) + \frac{i\Omega_s(z)}{2}(\sigma_{31}-\sigma_{13}) + \Gamma_2\sigma_{22} + \Gamma_3\sigma_{33} \\ v_z \frac{d}{dz}\sigma_{22} = -\frac{i\Omega_p(z)}{2}(\sigma_{21}-\sigma_{12}) - \Gamma_2\sigma_{22} \\ v_z \frac{d}{dz}\sigma_{33} = -\frac{i\Omega_s(z)}{2}(\sigma_{31}-\sigma_{13}) - \Gamma_3\sigma_{33} \end{cases}$$

$$\left\{ v_z \frac{d}{dz}\sigma_{21} = \frac{i\Omega_p(z)}{2}(\sigma_{11}-\sigma_{22}) - \frac{i\Omega_s(z)}{2}\sigma_{23} + (i\Delta_p - \gamma_{21})\sigma_{21} \right. \quad (7)$$

$$\left\{ v_z \frac{d}{dz}\sigma_{31} = \frac{i\Omega_s(z)}{2}(\sigma_{11}-\sigma_{33}) - \frac{i\Omega_p(z)}{2}\sigma_{32} + (i\Delta_s - \gamma_{31})\sigma_{31} \right.$$

$$\left\{ v_z \frac{d}{dz}\sigma_{32} = \frac{i\Omega_s(z)}{2}\sigma_{12} - \frac{i\Omega_p(z)}{2}\sigma_{31} + \big(i(\Delta_s - \Delta_p) - \gamma_{32}\big)\sigma_{32} \right.$$

Here $\Omega_s(z)$ and $\Omega_p(z)$ are the Rabi frequencies of the probe and pump respectively. Both are exponentially decaying along Z due to the evanescent electromagnetic field. σ_{ij} is the slowly varying density matrix element. The detunings, and lifetimes are defined as follows:

$$\Gamma_i = \begin{cases} \Gamma_0 + |k_z v_z| & i \neq 1 \\ 0 & i = 1 \end{cases}$$

$$\gamma_{ij} = \frac{\Gamma_i+\Gamma_j}{2} \qquad (8)$$

$$\Delta_s = \delta - \mathrm{k_x} \cdot \mathrm{v_x} \qquad \Delta_p = -\mathrm{k_x} \cdot \mathrm{v_x}$$

Where δ is the detuning of the probe beam from resonance, k_z is the imaginary k-vector that describes the evanescent decay of the optical mode, and k_x is the real propagation wave number. Here we assume that both modes, namely the pump and probes modes, have nearly the same wavenumbers. As we assume that $\Omega_p \gg \Omega_s$, and thus $\sigma_{11} + \sigma_{22} \approx 1$ (and thus $\sigma_{33} \approx 0$) we can write the following set of equations:

$$\left\{ v_z \frac{d}{dz}\sigma_{22} = -\frac{i\Omega_p}{2}(\sigma_{21} - \sigma_{12}) - \Gamma_2\sigma_{22} \right.$$

$$\begin{cases} v_z \frac{d}{dz}\sigma_{21} = \frac{i\Omega_p}{2}(1 - 2\sigma_{22}) + (i\Delta_p - \gamma_{21})\sigma_{21} \\ v_z \frac{d}{dz}\sigma_{12} = \frac{-i\Omega_p}{2}(1 - 2\sigma_{22}) + (-(\mathrm{i}\Delta_p) - \gamma_{21})\sigma_{12} \end{cases} \qquad (9)$$

$$\left\{ v_z \frac{d}{dz}\sigma_{31} = \frac{i\Omega_s}{2}(1 - \sigma_{22}) - \frac{i\Omega_p}{2}\sigma_{32} + (i\Delta_s - \gamma_{31})\sigma_{31} \right.$$

$$\left\{ v_z \frac{d}{dz}\sigma_{32} = \frac{i\Omega_s}{2}\sigma_{12} - \frac{i\Omega_p}{2}\sigma_{31} + \left(i(\Delta_s - \Delta_p) - \gamma_{32}\right)\sigma_{32} \right.$$

To find such solutions, we solve $\sigma_{13}(z, v\ )$ numerically, given an exponential decaying Ω_s and Ω_p, and an initial condition of $\sigma_{11} = 1$. For the steady state solution, we solve analytically the steady state (setting the derivatives to zero) set of equations.

In figure supplementary 4 we plot an example of such solutions, for the atoms moving along the z axis, i.e. those which satisfy V_x=0, and for a pump Rabi frequency of ~3GHz. We do so for two different velocities: in figure supplementary 4 we plot the probe σ_{31} coherence, as a function of distance from the surface for incoming and outgoing atoms with the speeds of -10m/s and +10m/s respectively (figure supplementary 4a), and for atoms having incoming and outgoing speeds of -200m/s and 200m/s respectively (figure supplementary 4b). As can be seen, such solutions differ both qualitatively, and quantitatively. For the slower atoms, the outgoing atoms experience a transient response, and undergo decaying Rabi oscillations, whereas the incoming atoms change their absorption in an adiabatic manner, up to a point where they start experiencing a strong pump beam (near the surface), and the induced transparency window starts to be evident. For the faster atoms, the outgoing atoms do not have sufficient time to develop Rabi oscillations, whereas the incoming atoms do not have sufficient time to be effected by the pump beam. Generally, in the case of a single photon experiment it has been pointed out, that both atomic groups have an equal contribution to the susceptibility. In contrast, as has been pointed by ref. 10 ,in our two photon scenario the two atomic groups do not contribute equally.

Finally, we plot the normalized susceptibility (given by Eq. 6). In figure supplementary 5, we plot the solution for two cases. First, in figure supplementary 5a, we plot the solution for varying pump powers. As can be clearly seen the probe susceptibility is strongly influenced by the presence of the pump, and a transit time broadened transparency window is created in

the center of susceptibility peak. Such results resemble the experimental results presented in the main article. Indeed we are able to nearly "shut down" the probe peak both in simulation and in the experiment. We note that the low pump power dips in the simulation are narrower than those measured. This is due to the fact that we have used a one-dimensional model, with a one dimensional transit time broadening, whereas our waveguide system is essentially two dimensional in respect to the transit time broadening. A two dimensional transit time broadening model shall broaden these lines significantly, as measured.

Finally, in order to verify that the strong dip within the susceptibility peak is indeed a coherent effect, we numerically "turn off" the coherent effects by nulling the σ_{32} coherence matrix element and plot again the probe susceptibility under similar pump conditions (figure supplementary 5b). As can be clearly seen, now we are unable to reduce the probes susceptibility to below 50 percent of its maximal value. In general, in absence of σ_{32} coherence, the only mechanisms to reduce of the probes susceptibility are atomic saturation and optical pumping. The absence of optical pumping discussed and justified in the main manuscript, and simulated here, leaves the saturation as the only mechanism for the reduction in the probe's susceptibility. As a consequence, in the absence of coherence, we are unable to achieve larger than 50 percent reduction in the susceptibility. As evident in the main text, the obtained contrast approaches 100% and thus coherent effects must contribute significantly. We note, that the small modulation dips that are evident at low pump powers in figure supplementary 5b are a consequence of a velocity selection saturation effect. This effect diminishes as power broadening increases.

<u>Supplementary Figure 3</u>:

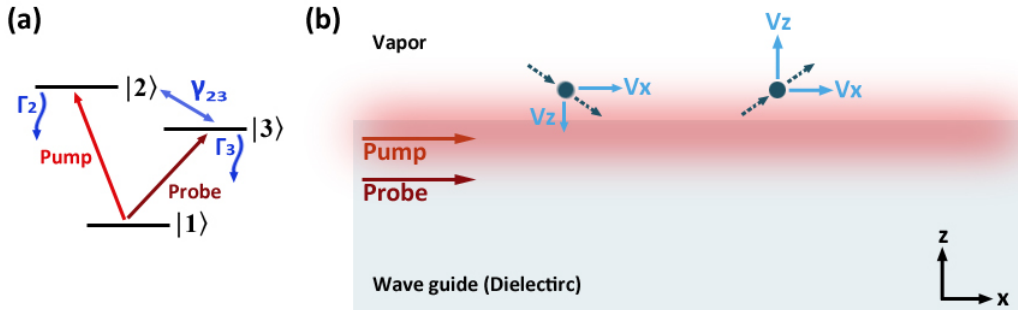

Supplementary Figure 3 | Level scheme and atoms in evanescent field illustration a) V-type atomic level scheme b) Illustration of an atom entering the evanescent region of a one dimensional wave guide, with an negative velocity Vz, and an atom exiting the evanescent region with a positive velocity Vz.

<u>Supplementary Figure 4</u>:

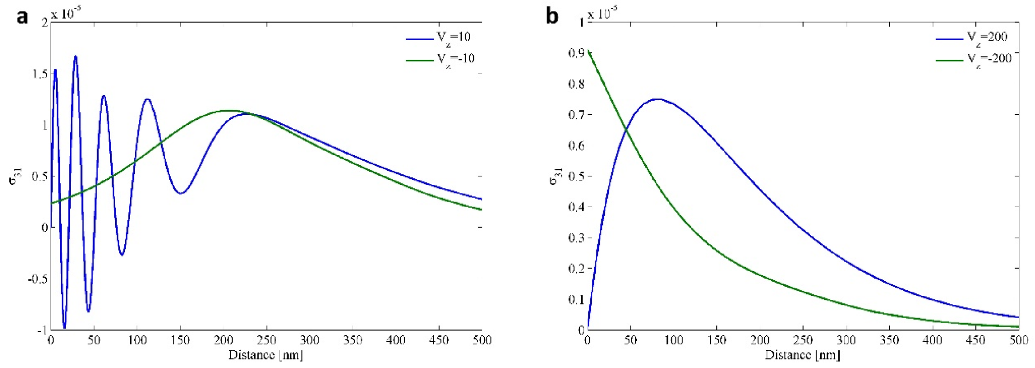

Supplementary Figure 4 | Calculated coherences for different velocity groups: a) σ_{31} as function of distance from surface for the incoming group of atoms with speed of -10 m/s and outgoing speeds of 10m/s b) σ_{31} as function of distance from surface for the incoming group of atoms with speed of -200 m/s and outgoing speeds of 200m/s

<u>Supplementary Figure 5</u>:

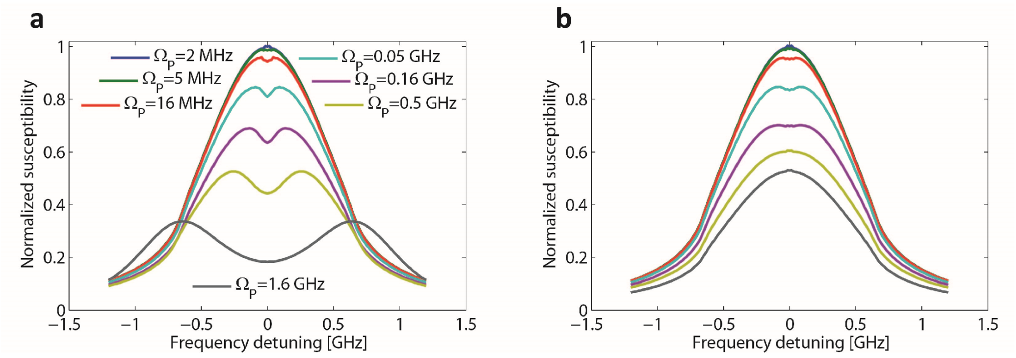

Supplementary Figure 5 | evanescent v-type pump-probe susceptibility a) Normalized atomic susceptibility as a function of frequency of evanescent V-type pump-probe excitation. The different curves correspond to varying pump powers. b) Normalized atomic susceptibility as function of frequency of evanescent V-type pump-probe excitation, where the coherence between levels 3 and 2 is set to zero.

<u>Supplementary note 4.</u>

Calculation of the Rabi frequencies

The purpose of this appendix is to present the calculations of the measured and theoretical Rabi frequencies presented in the main manuscript. First, we calculate the Rabi frequency as a function of the optical power in the wave guide.

Starting from the definition of Rabi frequency, and relating it to optical power:

$$|\Omega_r| = \left|\frac{d_{ij}\cdot E_0}{\hbar}\right| = \left(\frac{I}{2\epsilon_0 c}\right)^{\frac{1}{2}}\frac{d_{ij}}{\hbar} = \left(\frac{P/A}{2\epsilon_0 c}\right)^{\frac{1}{2}}\frac{d_{ij}}{\hbar} \qquad (10)$$

Where, d_{ij} is the dipole element, for a transition between levels F_i and F_j, ϵ_0 is the permittivity, c the speed of light, I is the optical intensity, P the optical power and A the area of the optical mode. Considering a mode area of 1200X250 nm^2, and taking the typical optical dipole element of the F=2 to F'=1/2/3 to be $1.1\cdot 10^{-29}C\cdot m$ we obtain:

$$\frac{\Omega_r}{2\pi\sqrt{P}} = 419 \quad \left[MHz/\sqrt{\mu W}\right] \qquad (11)$$

Next, we relate the measured slope in Fig. 4b to a Rabi frequency, using the following relation:

$$\Delta f = \frac{1}{8\pi}\frac{\Omega_r^2}{\Delta\omega} = \frac{1}{8\pi}\frac{\alpha}{\Delta\omega}P \qquad (12)$$

Where Δf is the light shift, $\Omega_r^2 = \alpha P$ and $\Delta\omega$ is the detuning. Recalling that our measured slope was 13.49 $MHz/\mu W$, we obtain

$$\alpha = 6.47\cdot 10^6\frac{MHz^2}{\mu W} \quad \rightarrow \quad \frac{\Omega_r}{2\pi\sqrt{P}} = \frac{\sqrt{\alpha}}{2\pi} = \left[405\ MHz/\sqrt{\mu W}\right] \qquad (13)$$

Supplementary References:

1. Nienhuis, G., Schuller, F. & Ducloy, M. Nonlinear selective reflection from an atomic vapor at arbitrary incidence angle. *Phys. Rev. A* **38,** 5197–5205 (1988).
2. Guo, J., Cooper, J., Gallagher, A. & Lewenstein, M. Theory of selective reflection spectroscopy. *Opt. Commun.* **110,** 197–208 (1994).
3. Zhao, K. & Wu, Z. Regionally specific hyperfine polarization of Rb atoms in the vicinity (□ 10^{-5} cm) of surfaces. *Phys. Rev. A* **71,** 012902 (2005).
4. Kondo, R., Tojo, S., Fujimoto, T. & Hasuo, M. Shift and broadening in attenuated total reflection spectra of the hyperfine-structure-resolved D 2 line of dense rubidium vapor. *Phys. Rev. A* **73,** 062504 (2006).
5. Stern, L., Desiatov, B., Goykhman, I. & Levy, U. Nanoscale light–matter interactions in atomic cladding waveguides. *Nat. Commun.* **4,** 1548 (2013).
6. Ritter, R. *et al.* Atomic vapor spectroscopy in integrated photonic structures. *Appl. Phys. Lett.* **107,** 041101 (2015).
7. Stern, L., Grajower, M. & Levy, U. Fano resonances and all-optical switching in a resonantly coupled plasmonic–atomic system. *Nat. Commun.* **5,** (2014).
8. Bordé, C. J., Hall, J. L., Kunasz, C. V. & Hummer, D. G. Saturated absorption line shape: Calculation of the transit-time broadening by a perturbation

approach. *Phys. Rev. A* **14,** 236–263 (1976).
9. Whittaker, K. A. *et al.* Optical response of gas-phase atoms at less than λ/80 from a dielectric surface. *Phys. Rev. Lett.* **112,** 253201 (2014).
10. Schuller, F., Gorceix, O. & Ducloy, M. Nonlinear selective reflection in cascade three-level atomic systems. *Phys. Rev. A* **47,** 519–528 (1993).
11. Meng, T. *et al.* V-type electromagnetically induced transparency and saturation effect at the gas-solid interface. 7 (2015). at <http://arxiv.org/abs/1503.04950>
12. Sen, S., Dey, T. K., Nath, M. R. & Gangopadhyay, G. Comparison of electromagnetically induced transparency in lambda, cascade and vee three-level systems. *J. Mod. Opt.* (2015). at <http://www.tandfonline.com/doi/abs/10.1080/09500340.2014.960019>
